# Comparison of Diffusion MRI Acquisition Protocols for the *In Vivo* Characterization of the Mouse Spinal Cord: Variability Analysis and Application to an Amyotrophic Lateral Sclerosis Model

**DOI:** 10.1371/journal.pone.0161646

**Published:** 2016-08-25

**Authors:** Matteo Figini, Alessandro Scotti, Stefania Marcuzzo, Silvia Bonanno, Francesco Padelli, Victoria Moreno-Manzano, José Manuel García-Verdugo, Pia Bernasconi, Renato Mantegazza, Maria Grazia Bruzzone, Ileana Zucca

**Affiliations:** 1 Scientific Direction, Fondazione IRCCS Istituto Neurologico “Carlo Besta” Milan, Italy; 2 Neurology IV—Neuroimmunology and Neuromuscular Diseases Unit, Fondazione IRCCS Istituto Neurologico “Carlo Besta”, Milan, Italy; 3 Neuronal and Tissue Regeneration Laboratory, Centro de Investigación Príncipe Felipe, Valencia, Spain; 4 Unidad de Neurobiología comparada, Universidad de Valencia, Valencia, Spain; 5 Neuroradiology, Fondazione IRCCS Istituto Neurologico “Carlo Besta”, Milan, Italy; Uniformed Services University, UNITED STATES

## Abstract

Diffusion-weighted Magnetic Resonance Imaging (dMRI) has relevant applications in the microstructural characterization of the spinal cord, especially in neurodegenerative diseases. Animal models have a pivotal role in the study of such diseases; however, in vivo spinal dMRI of small animals entails additional challenges that require a systematical investigation of acquisition parameters. The purpose of this study is to compare three acquisition protocols and identify the scanning parameters allowing a robust estimation of the main diffusion quantities and a good sensitivity to neurodegeneration in the mouse spinal cord. For all the protocols, the signal-to-noise and contrast-to noise ratios and the mean value and variability of Diffusion Tensor metrics were evaluated in healthy controls. For the estimation of fractional anisotropy less variability was provided by protocols with more diffusion directions, for the estimation of mean, axial and radial diffusivity by protocols with fewer diffusion directions and higher diffusion weighting. Intermediate features (12 directions, b = 1200 s/mm^2^) provided the overall minimum inter- and intra-subject variability in most cases. In order to test the diagnostic sensitivity of the protocols, 7 G93A-SOD1 mice (model of amyotrophic lateral sclerosis) at 10 and 17 weeks of age were scanned and the derived diffusion parameters compared with those estimated in age-matched healthy animals. The protocols with an intermediate or high number of diffusion directions provided the best differentiation between the two groups at week 17, whereas only few local significant differences were highlighted at week 10. According to our results, a dMRI protocol with an intermediate number of diffusion gradient directions and a relatively high diffusion weighting is optimal for spinal cord imaging. Further work is needed to confirm these results and for a finer tuning of acquisition parameters. Nevertheless, our findings could be important for the optimization of acquisition protocols for preclinical and clinical dMRI studies on the spinal cord.

## Introduction

Diffusion-weighted Magnetic Resonance Imaging (dMRI) [1] has an essential role in routine clinical MRI, mainly for its sensitivity to microstructural alterations in many pathologies. The most widely applied dMRI technique is Diffusion Tensor Imaging, (DTI), which characterizes the three-dimensional tissue microstructure [2,3] with the diffusion tensor, usually summarized by the derived parameters: mean diffusivity (MD), fractional anisotropy (FA) [4,5] and, in cylindrically anisotropic conditions, axial (AD) and radial diffusivity (RD) [6].

In particular, DTI has been shown to be useful in the spinal cord (SC) to inquire a number of different pathologies, especially those involving white matter, in both patients and animal models. Several studies demonstrated the utility of SC DTI in patients with SC traumatic injury (SCI), cervical spondylotic myelopathy, SC tumors, multiple sclerosis and amyotrophic lateral sclerosis (ALS) [7,8].

In animal models of SCI, the AD has been shown to rapidly decrease in the site of traumatic injury [9]. In demyelinating disease models [10–12], both a reduction of AD and an increase in RD were found. Considering ALS mice, the few DTI studies present in literature reported a reduction of FA and AD and less significant results on MD and RD values in the spinal cord [13,14].

However, high-field SC DTI studies on small animals imply some technical challenges to avoid several artifacts in Echo Planar Imaging (EPI) sequences, mainly related to respiratory motions, field inhomogeneity and off-centering of the SC in the axial plane [15].

Furthermore, the diffusion tensor is not a perfect model of water diffusion in biological systems [16,17] and its estimation, based on signal loss, is inherently sensitive to noise. Consequently, DTI-derived quantities have been shown to depend on signal-to-noise ratio (SNR) and acquisition parameters, such as the b-value and the number of diffusion-encoding directions. In particular, diffusivities reduce with the b-value [18,19], diffusion anisotropy increases at low SNR [20] and the estimation variance of all the parameters decreases with the number of diffusion-encoding directions [21]. Changing any of these acquisition parameters has an effect on the acquisition time, which must be kept reasonably short in *in vivo* experiments. Therefore, the choice of the optimal *in vivo* protocol with maximum sensitivity to the microstructural features of interest in the studied tissue, requires a time constraint.

Several studies investigated the optimal acquisition parameters for a robust estimation of the diffusion tensor with analytical and empirical methods [22–26], but it is not always clear how to transfer these numerical results into practice, where additional sources of noise and variability are present [27]. Moreover, most of these studies are based on the hypothesis that fiber directions are randomly distributed, whereas in the SC all the fibers are mainly oriented along the spinal axis. For example, Jones [21] suggested a scheme with at least 30 directions to avoid biases related to the different local orientations, but in a coherently ordered structure as the SC these biases would be certainly less important than in a complex environment as the brain.

An a posteriori approach alternative to numerical analysis is to evaluate the reproducibility and noisiness of DTI in healthy conditions, either qualitatively, assessing the visual contrast in DTI maps [28], or quantitatively, measuring the variability among different measures in control groups [29]. This may have a more limited impact because it requires the acquisition of real data in a necessarily finite number of conditions, but has the advantage of evaluating the ultimate output of diffusion without any a priori assumption. However, the comparison of SC DTI acquisition protocols was performed only for humans [28,29] and not for small animals.

Indeed, all the *in vivo* DTI studies on mouse SC used only the minimum 6 gradient directions needed for the estimation of the tensor [9–15,30–35], with only one exception applying 12 directions [36]. This is probably due to technical difficulties and to keep the acquisition time short when using non-EPI sequences; indeed SC studies on the rat, which are advantageous given the larger dimension of the spine, show a larger variability and complexity of acquisition protocols [37–48]. Moreover, a study suggested that measuring diffusion only along the spine and in the perpendicular direction gives similar information to the full tensor [49] and this approach was followed by some groups [50,51].

To our knowledge, the optimization of diffusion sampling scheme for the spinal cord has never been thoroughly investigated in a quantitative way, though acquisition and reconstruction strategies have been evaluated and compared [15, 35], this issue seems to have been neglected.

In the present work we compare three different *in vivo* dMRI protocols applied to investigate the microstructure of both gray (GM) and white matter (WM) in the lumbar region of the mouse spinal cord.

The three protocols were chosen to probe three different sampling strategies for dMRI with the constraint of an acquisition time of about 35 minutes: the first protocol has a very high directional resolution; the second one explores a lower number of diffusion directions, with a higher b-value but a higher SNR in each volume due to the higher number of repetitions; the third one acquires data along two directions only, but with multiple b-values.

In the first stage of the study, we compared the protocols in wild type mice considered as healthy controls, measuring the variability between and within the animals and evaluating the differences in the estimated parameters among the protocols. In a second stage, we compared the performance of the three protocols in the detection and characterization of microstructural SC lesions in G93A-SOD1 transgenic mouse, an ALS animal model. This model over-expresses a mutated form of the human SOD1 gene and presents symptoms and neuropathological features that mimic those characteristic of familial ALS [52].

## Materials and Methods

### Animals

Transgenic G93A-SOD1 (B6SJL-Tg(SOD1*G93A)1Gur) mice carrying a high-copy number of mutant human allele SOD1 and transgenic WT-SOD1 (B6SJL-Tg(SOD1)2Gur/J) mice were purchased from Charles Rivers Laboratories International, Inc. (Wilmington, MA, USA), maintained and bred at the animal house of the Besta Institute according to the institutional guidelines and international laws (EEC Council Directive 86/609, OJL 358, 1, December 12, 1987, NIH Guide for the Care and Use of Laboratory Animals, U.S. National Research Council, 1996). In particular mice were housed in ventilated cages (3–5 animals for each cage); water and food were given ad libitum. Material (as sterilized paper and cardboard chips) was provided to the animals for the construction of the nest as environmental enrichment. The day / night cycle was of 12:12 hours per day; the temperature of the livestock buildings was of 22 ± 2°C. The animals have been checked daily to assess the level of well-being and health. Animal studies were approved by the Italian Ministry of Health (codes: IMP-01-12). Animals were sacrificed by exposure to carbon dioxide. Male mice were used in all the studies. G93A-SOD1 and WT-SOD1 progenies were identified by real-time polymerase chain reaction of the human SOD1 gene, as previously described [53].

### MRI Setup

MRI experiments were performed on 7 G93A-SOD1 and 7 WT-SOD1 mice at two time points: 10 and 17 weeks of age, corresponding to the asymptomatic and symptomatic stage of disease [52].

The acquisitions were carried out on a 7 T MRI scanner (Bruker BioSpec 70/30, Ettlingen, Germany) equipped with a gradient system reaching the maximum amplitude of 440 mT/m. Mice were anaesthetized with 1.5–2% isoflurane (flow rate 0.8 L/min) and positioned on an animal bed equipped with a nose cone for gas anesthesia. To detect the depth of anesthesia and the animal health condition during the MRI study, the respiratory rate was monitored by a pneumatic sensor. A 75 mm birdcage linear coil (Rapid MR International, Ohio, USA) was used for radio frequency excitation and a rat brain surface coil (Rapid) was used for signal reception. A custom setup was implemented to obtain the optimal visualization of the anatomical district of interest: animals were placed supine on the rat brain surface coil in order to position the lumbar tract of the spine at the center of the most sensitive part of the coil. The supine position of the mouse on the surface coil also helped reduce the respiratory motion of the spine without need of respiratory trigger: preliminary experiments highlighted that the movement reduction due to respiratory triggering was negligible if compared to the increase of acquisition time.

Fast T2-weighted images were acquired as anatomical references in three orthogonal planes: axial, sagittal, and coronal. The correct animal alignment in the magnet bore was verified by these scans.

### Diffusion MRI

Three different diffusion acquisition protocols were applied. The first one (protocol A) consisted of a single-shot EPI sequence with the following parameters: 126 diffusion-encoding gradient directions (Ndir), b = 700s/mm^2^, δ = 3 ms, Δ = 11 ms, TE = 22.3 ms, TR = 2100 ms, number of volumes without diffusion weighting (Nb0) = 10, number of averages (NA) = 6, number of repetitions (NR) = 1.

The second protocol (B) consisted of a single-shot EPI sequence with Ndir = 12, b = 1200 s/mm^2^, δ = 4 ms, Δ = 11 ms, TE = 23.3 ms, TR = 2100 ms, Nb0 = 5, NA = 4, NR = 15.

The third protocol (C) was an EPI sequence with Ndir = 2 (the slice and the read direction, approximately parallel and perpendicular to the axis of the spinal cord), b = 200, 400, 800, 1200, 1500 s/mm^2^, δ = 4 ms, Δ = 11 ms, TE = 23.3 ms, TR = 2100 ms, Nb0 = 5, NA = 4, NR = 20. In each scan, 8 contiguous axial slices of the spinal cord at the lumbar level with slice thickness = 0.8 mm, in-plane resolution = 0.109 x 0.078 mm^2^, FOV = 1.4 x 1 cm^2^ were obtained.

**Fig 1:**
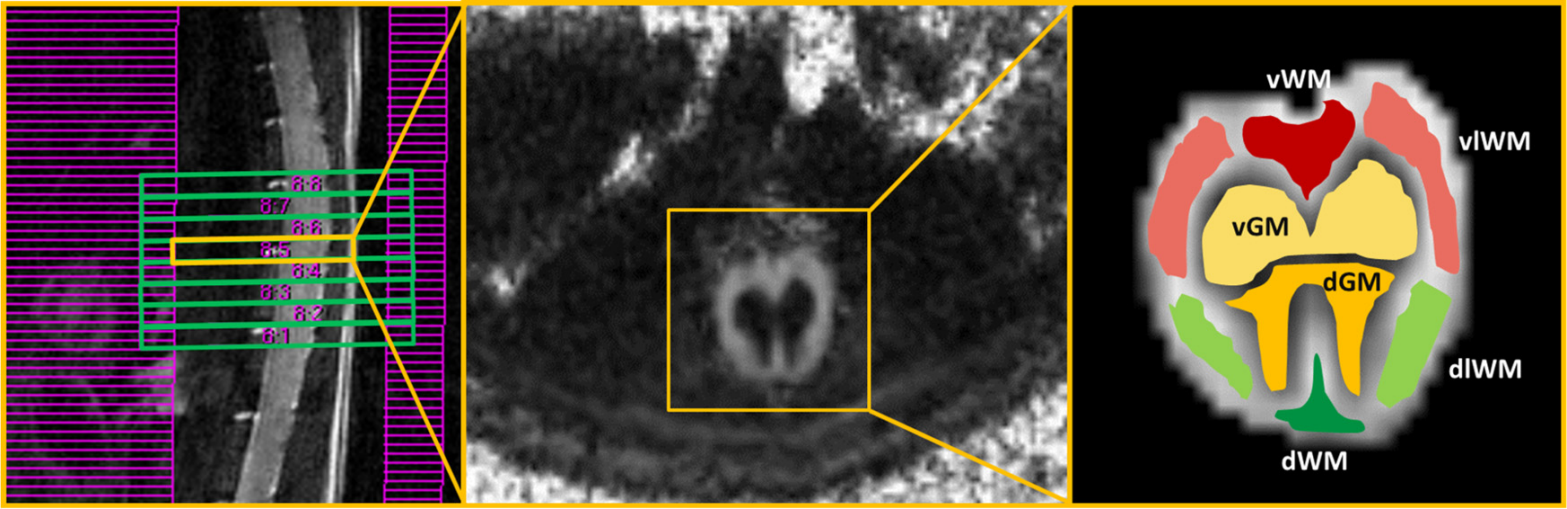
Acquisition setup and ROI delineation. Left: T2-weighted sagittal view of the lumbar tract. The green boxes are the image slices and the purple grid shows the position of the saturation slices. Note that the examined lumbar spinal cord region is mostly straight and the imaging slices were placed perpendicular to the axis of the spine. Center: FA map of a single slice (framed in yellow on the left panel). Right: ROIs overlaid on a part of the FA map including the whole section of the spinal cord (in yellow on the central panel); ventral (vWM), dorsal (dWM), ventro-lateral (vlWM) and dorso-lateral (dlWM) white matter, ventral (vGM) and dorsal (dGM) gray matter.

The acquisition time was about 35 minutes for each protocol. The magnetic field homogeneity was optimized by a localized second order shimming procedure featured on a volume of interest covering the whole field of view by using the Mapshim macro (Paravision 5.1, Bruker). Saturation slices were placed as shown in Fig 1 to avoid folding artifacts.

In a preliminary phase, the readout bandwidth was optimized in all the protocols in order to keep an acceptable tradeoff between SNR and spatial distortions [54], but also to minimize Nyquist ghosts, which can affect DTI results [55]. All images were corrected for motion and distortions with an affine 2D registration to the b0 volume in FSL (FMRIB Software Library) using FLIRT (FMRIB's Linear Image Registration Tool) [56]; the registration was limited to in-plane corrections as in [57], because the expected animal motions are perpendicular to the spine, and the spinal cord appearance is similar at different levels, hence a 3D registration process could result in misalignment along the direction of the spinal axis. The registered volumes were averaged across repetitions for protocols B and C.

For protocols A and B, a set of diffusion parameters (FA, MD, AD and RD) was obtained from the tensor estimation performed by a home-made code developed in MatLab (The Mathworks, Natick, MA, USA). For protocol C, the apparent diffusion coefficient was estimated in both directions by a home-made MatLab code, and the diffusion quantities derived according to the following expressions:

$${AD}_{C}={ADC}_{s}$$

$${RD}_{C}={ADC}_{p}$$

$${MD}_{C}=\frac{{ADC}_{s}+2∙{ADC}_{r}}{3}$$

$${FA}_{C}=\sqrt{\frac{3}{2}∙\frac{{\left({ADC}_{s}-{MD}_{C}\right)}^{2}+2{\left({ADC}_{r}-{MD}_{C}\right)}^{2}}{{{ADC}_{s}}^{2}+2∙{{ADC}_{r}}^{2}}}$$

where *ADC*_*s*_ and *ADC*_*r*_ are the estimated apparent diffusivities along the slice direction, approximately corresponding to the direction of the spinal cord, and along the read direction, perpendicular to it.

Six regions of interest (ROIs) were manually outlined by an experienced radiologist on all the eight slices of the FA maps. They included: ventral, dorsal, ventro-lateral and dorso-lateral white matter (vWM, dWM, vlWM, dlWM), ventral and dorsal gray matter (vGM, dGM), as shown in Fig 1.

### Statistical Analysis

The SNR of the raw images from each acquisition protocol was evaluated as follows:

$${SNR}_{WM}=\frac{{MI}_{WM}}{{SD}_{BG}}$$

$${SNR}_{GM}=\frac{{MI}_{GM}}{{SD}_{BG}}$$

where *MI*_*WM*_ and *MI*_*GM*_ are the mean signal intensities in a ROI covering the whole WM and in a ROI covering the whole GM, respectively, and *SD*_*BG*_ is the standard deviation in a ROI placed in the background.

The contrast-to-noise ratio (CNR) between WM and GM for the map of each DTI metric was computed as:

$$CNR=\frac{M_{WM}-M_{GM}}{\sqrt{\frac{{{SD}_{WM}}^{2}+{{SD}_{GM}}^{2}}{2}}}$$

where *M*_*WM*_ and *SD*_*WM*_ are the mean and standard deviation of the considered parameter in a region of interest covering the whole WM, and *M*_*GM*_ and *SD*_*GM*_ are the mean and standard deviation in a region of interest covering the whole GM.

The variability of imaging measurements was evaluated inter- and intra-subject by coefficient of variation (CV) computation. CV is defined as the ratio between the standard deviation of a set of measurements and its mean [58]. For the inter-subject CV we computed the mean values of the parameters within each ROI and divided the standard deviation of these mean values by their average. Similarly, the mean value and standard deviation of each parameter within each ROI, resulted in an individual intra-subject CV, eventually averaged among the mice.

The statistical significance of the differences in intra-subject CV and mean values among the three protocols was assessed by a two-sided Mann-Whitney test with a significance level of p < 0.05 for each parameter in each ROI of WT-SOD1 mice at the two time points.

To evaluate the effect of the approximation made in protocol C, which considers the slice direction as the direction of the fibers, we computed the angle between the slice direction and the main diffusion direction estimated from the tensor in protocols A and B as:

$$\vartheta =arccos(v_{1,s})$$

where *v*_1,*s*_ is the component of the first eigenvector of the diffusion tensor along the slice direction.

The deviation between the tensor-derived quantities and those obtained by considering the tensor component along the axes of the imaging frame of reference was computed as well. Finally, the correlation between the deviation angles and the parameters derived from protocol C in the corresponding ROIs of the same mice was assessed by the Spearman's rank correlation coefficient.

The performance of each protocol in differentiating the two groups was evaluated by the effect size (ES) [59], which was defined as the difference between the mean values in the two groups divided by the standard deviation of the WT-SOD1 group.

The differences between the parameters estimated at 10 and 17 weeks of age within the two groups were evaluated in the same way. The statistical significance of all these differences was also evaluated by a two-sided Mann-Whitney test with a significance level of p < 0.05.

Statistical analyses were performed in MatLab.

### Histology and Transmission Electron Microscopy (TEM)

Lumbar spinal cord tissues were obtained from two WT-SOD1 and two G93A-SOD1 mice at 10 and 17 weeks of age. Mice were perfused with physiologic saline (0.9% NaCl) and fixation solution (2% of paraformaldehyde and 2% of glutaraldehyde), and the dissected lumbar sections were post-fixed for additional 4 hr. After washes, sections were fixed with 2% osmium, rinsed, dehydrated and embedded in Durcupan resin (Fluka, Sigma-Aldrich, St. Louis, USA). Semi-thin sections (1.5 μm) were cut with an Ultracut UC-6 and stained lightly with 1% toluidine blue used for histological analysis. Ultra-thin sections (0.08 μm) were cut with a diamond knife, stained with lead citrate (Reynolds solution) and examined under a transmission electron microscope FEI Tecnai G2 Spirit (FEI Europe, Eindhoven, Netherlands) attached to a digital camera Morada (Olympus Soft Image Solutions GmbH, Münster, Germany).

## Results

### Image Quality, SNR and CNR

Visual inspection of the acquired images revealed the absence of evident artifacts in the region of the spinal cord. The SNR of the raw images was always greater than 10 both in WM and in GM, except for the diffusion-weighted volumes of protocols B and C before averaging; however, after motion correction and averaging the SNR of protocols B and C was much higher than in protocol A.

The best CNR between WM and GM was provided by FA and AD, followed by RD; as expected, MD provided the lowest contrast between WM and GM (S1 Table).

### Mean Values of DTI Parameters

**Fig 2:**
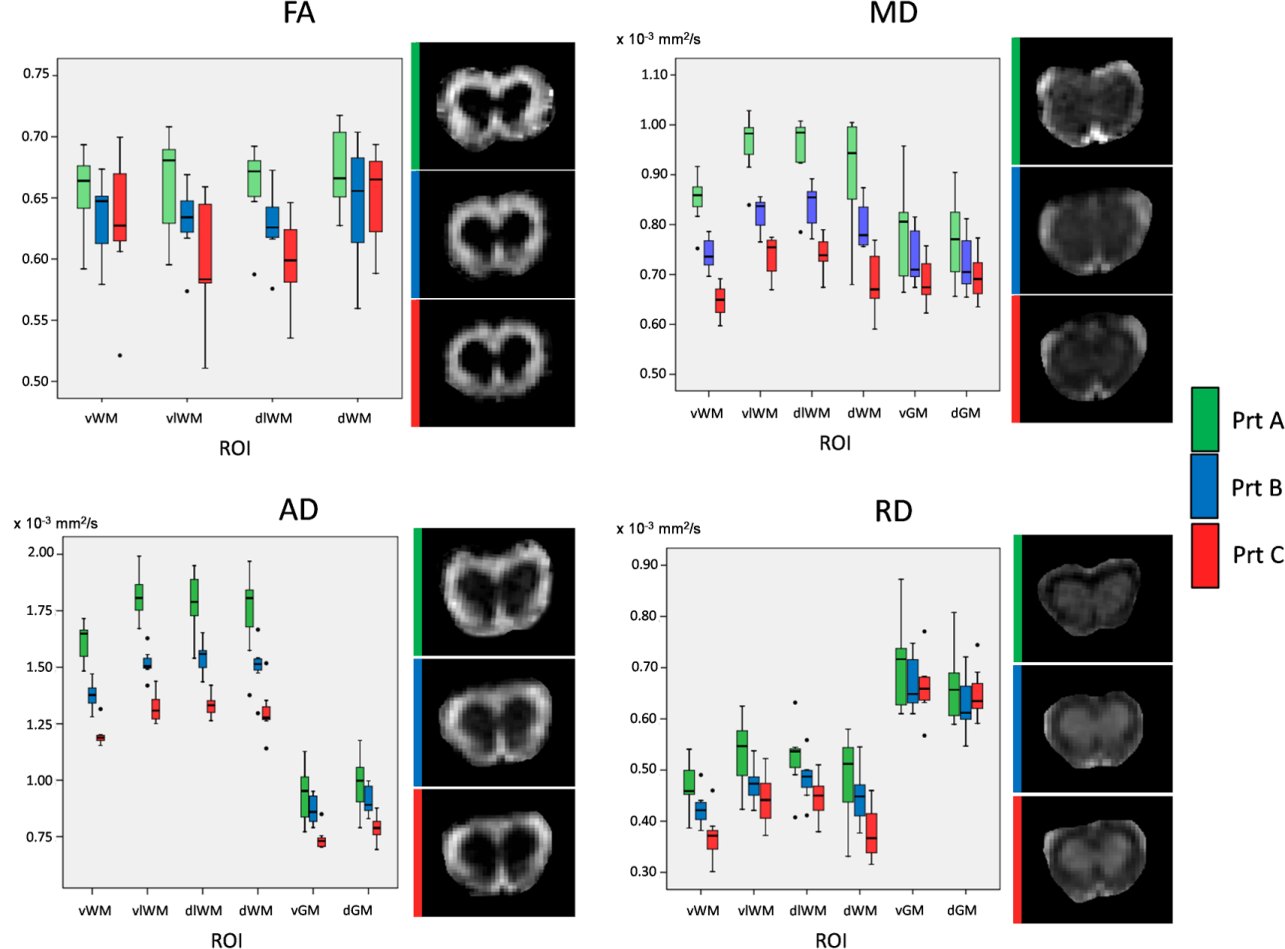
Mean values of DTI parameters and representative maps in healthy animals. Left side of each panel: box plots for the diffusion parameters estimated by each protocol in the 7 WT-SOD1 mice at week 10. Protocols are indicated by colors; the horizontal line corresponds to the median; the bottom and top limits of the boxes are the 1^st^ and 3^rd^ quartile; the ends of the whiskers are the lowest and highest value within 1.5 times the interquartile range from the first and third quartile respectively; isolated points are outliers. Right side of each panel: representative maps of the same parameter.

Mean values of the estimated DTI parameters follow a stepwise trend among the protocols, with protocol A providing the highest FA and diffusivities, followed by protocol B and protocol C (Fig 2). This tendency is similarly reproduced everywhere for the four diffusion quantities, with only some exceptions in which FA or RD were slightly higher in protocol C than in protocol B. Most of the FA and RD differences among protocols, though similar in all the ROIs, were not statistically significant; instead, differences in MD and AD were significant, with only few exceptions. The results obtained at 10 weeks of age are shown in Fig 2. FA in GM was not reported because the differences among the protocols are less important than in WM due to the smaller absolute values, and the evaluation of diffusion anisotropy in GM is less meaningful from a biological point of view.

### Inter-Subject CV

**Fig 3:**
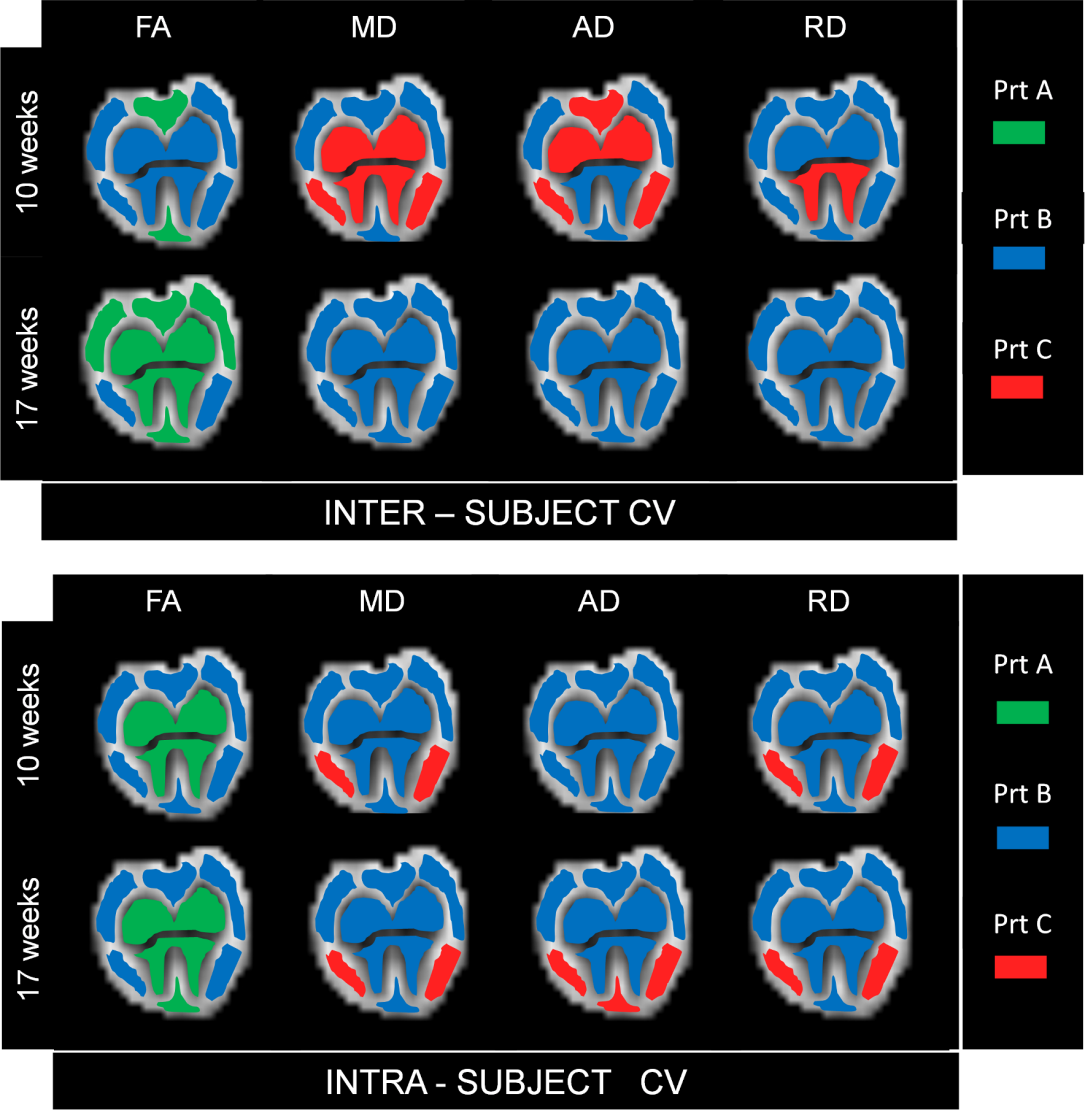
Inter-and intra-subject CV. Upper panel: The color in each ROI indicates the protocol with the lowest inter-subject coefficient of variation, thus the minimum variability of each DTI parameter (FA, MD, AD, RD) among animals at 10 and 17 weeks of age. Lower panel: The color in each ROI indicates the protocol with the lowest intra-subject coefficient of variation, thus the minimum variability of each DTI parameter (FA, MD, AD, RD) inside the ROI, averaged among animals at 10 and 17 weeks of age.

The comparison of the coefficients of variation among the 7 WT-SOD1 mice showed slightly different scenarios for anisotropy and diffusivities, depicted in the upper panel of Fig 3 with a color-coded representation showing the best protocol in each region.

Considering FA, protocols A and B had lower CVs than protocol C in all the ROIs, but they showed minor differences between themselves.

For diffusivities (MD, AD and RD), protocol B showed the lowest CV in most of the cases, followed by protocol C, while protocol A had generally the highest CV.

The differences between the CVs for protocols B and C were quite low, especially in GM and for AD also in WM; indeed, protocol C had a lower CV than protocol B in some cases at 10 weeks, but in all of them the difference was lower than 15%.

### Intra-Subject CV

The comparison of the coefficients of variability within the ROIs of every single mouse revealed an overall lower variability in protocol B, with some exceptions (lower panel of Fig 3).

The lowest CV for FA was obtained using protocol B in WM and protocol A in GM.

Considering diffusivity values, the CV was lower with protocol B than with protocol C in most of the cases, but the differences were generally quite low and not statistically significant. Protocol A showed higher CV than the others in all the ROIs for all the diffusivity parameters, with statistically significant differences in most of the regions.

### Angular Deviation

The mean angle between the slice direction and the main diffusion direction estimated by DTI was always similar for protocols A and B. In WM it extended from 7 to 14 degrees, with the maximum difference reported in dorso-lateral WM, whereas GM showed a larger deviation, up to 55 degrees (see S2 Table).

The relative difference between the parameters estimated by the eigenvalues of the diffusion tensor and by its components along the axes of the imaging frame of reference in WM were low. In particular, for MD it was always negligible and for AD it was less than 5%; for RD and FA it was less than 5% except in dorso-lateral WM, where the variation was between 7% and 9% (see S3 Table).

A significant but weak correlation between the angles and the parameters estimated by protocol C in WM was observed for FA, MD and RD; as expected, it was positive for RD and negative for FA. However, the coefficients were always lower than 0.5, showing a low dependence of DTI parameters on the angle between the slice direction and the main diffusion direction.

### Differences between G93A-SOD1 and WT-SOD1 Mice

At week 10, protocols A and B showed significant differences between G93A-SOD1 and WT-SOD1 mice for diffusivities in few ROIs (MD and RD in dlWM for protocol A; AD in dGM for protocol B), whereas protocol C revealed no significant difference.

At week 17, all the protocols showed lower FA, MD and AD in both GM and WM and lower RD in GM of G93A-SOD1 mice compared to WT-SOD1; for RD in WM, protocol A displayed lower values in G93A-SOD1 than in control mice, in contrast to protocols B and C that showed higher RD values in G93A-SOD1 than in WT-SOD1 mice.

Differences between the groups were statistically significant in most of the ROIs for protocols A and B, whereas protocol C revealed significant differences only in few cases (Table 1).

**Table 1:** Differences in DTI parameters between G93A-SOD1 and WT-SOD1 at week 17.

ROI	Protocol A				Protocol B				Protocol C			
	FA	MD	AD	RD	FA	MD	AD	RD	FA	MD	AD	RD
vWM	-4.14*	-1.75*	-3.03*	-0.13	-4.56*	-2.26*	-5.25*	1.18	-1.00	-0.75	-1.71*	0.29
vlWM	-3.27*	-2.15*	-3.86*	-0.26	-3.64*	-1.31	-5.65*	1.91*	-1.63*	-0.03	-2.16	1.01
dlWM	-3.06*	-2.14*	-3.86*	-0.45	-4.24*	-1.91	-7.74*	1.00	-1.36*	-0.72	-2.18*	0.48
dWM	-1.32	-2.12*	-2.85*	-1.05	-1.70*	-1.15	-1.75*	0.36	-0.68	-0.37	-0.77	0.36
vGM	-3.66*	-1.68*	-2.27*	-1.16*	-1.81*	-2.82*	-2.89*	-2.36*	-0.63	-0.98	-0.89	-0.80
dGM	-3.78*	-1.98*	-2.43*	-1.41*	-1.31*	-2.83*	-2.43*	-2.86*	-0.72	-1.11	-1.12	-0.92

The table includes the effect size for the comparison of each DTI parameter between G93A-SOD1 and WT-SOD1 mice. Statistically significant differences (p < 0.05) are marked by asterisks (*). In bold are the results associated with significant differences between the two groups for both protocols A and B.

**Fig 4:**
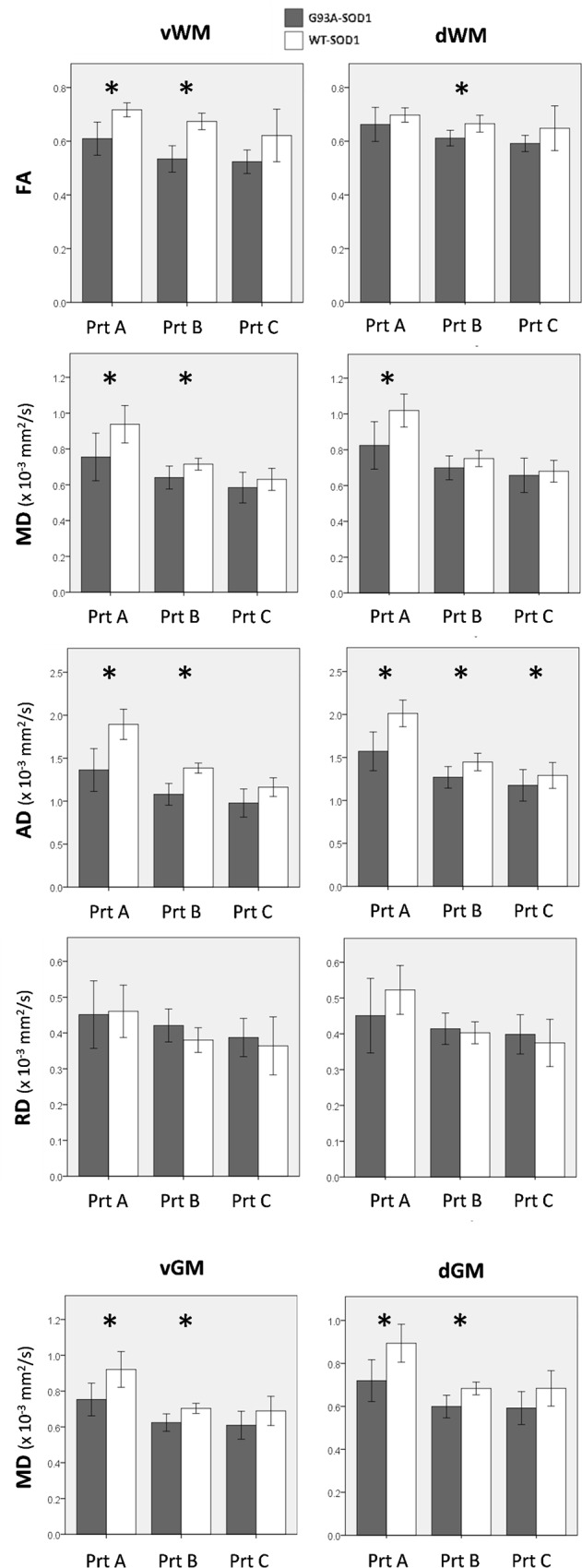
Comparison of DTI parameters between G93A-SOD1 and WT-SOD1 mice. The bar graphs show representative MRI quantities estimated in 7 WT-SOD1 (white) and 7 G93A-SOD1 mice (gray) at 17 weeks of age by each protocol (Prt). Mean values (± standard deviation) are shown for ventral (left) and dorsal (right) WM in the first 4 rows, for ventral (left) and dorsal (right) GM in the last row. Significant differences (p < 0.05) between G93A-SOD1 and WT-SOD1 are labeled with asterisks.

**Fig 5:**
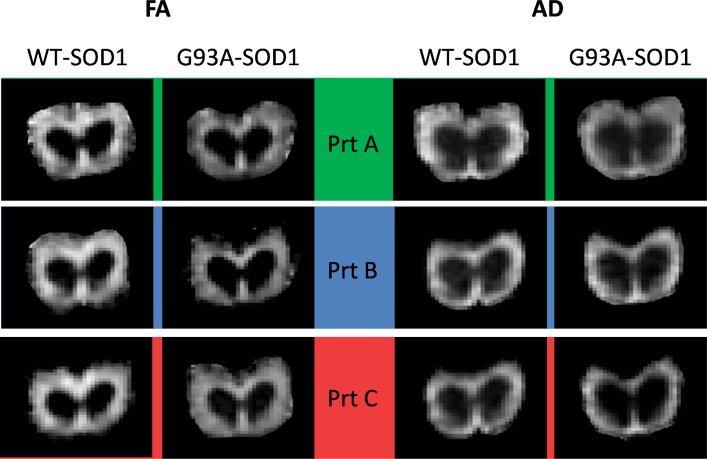
Representative FA and AD maps for 17-week old mice. The images show representative FA (left) and AD maps (right) for WT-SOD1 and G93A-SOD1 mice at 17 weeks of age. Each row correspond to a different protocol. Please note that the same contrast was used for all the FA and for all the AD maps, so that different intensities reflect different values of the parameters.

Representative comparisons between pathological and healthy animals with the three protocols are shown in Fig 4. The complete set of bar graphs for the comparison of each DTI parameter at 17 week of age is available in S1–S4 Figs. Representative FA and AD maps for WT-SOD1 and G93A-SOD1 mice are shown in Fig 5, showing a widespread reduction of both the parameters in diseased animals compared to age-matched controls.

The ES values for all the comparisons between G93A-SOD1 and WT-SOD1 mice at 17 weeks of age are reported in Table 1. Considering the regions where both protocols A and B showed statistically significant differences between the two groups (bold in Table 1), protocol B resulted in a higher ES in 12 cases out of 16. Considering the 4 cases in which protocol C showed significant differences, all the associated ES values were lower than those for protocols A and B.

### Monitoring the Pathological Changes over Time

In G93A-SOD1 mice at 17 weeks of age all the protocols found generally decreased FA, MD and AD with respect to the same animals at 10 weeks of age. They also found decreased RD in GM, whereas RD in WM was decreased for protocol A and generally increased for protocols B and C (Table 2). Most of these differences were in accordance to those found between G93A-SOD1 and WT-SOD1 animals at week 17, but smaller and less significant (Table 2).

**Table 2:** Differences in DTI parameters of G93A-SOD1 mice between 10 and 17 weeks of age.

ROI	Protocol A				Protocol B				Protocol C			
	FA	MD	AD	RD	FA	MD	AD	RD	FA	MD	AD	RD
vWM	-1.22	-1.36	-1.90*	-0.53	-2.59*	-1.07	-2.36*	0.60	-1.29*	-0.49	-1.36*	0.47
vlWM	-1.17	-1.26	-1.69*	-0.39	-2.62*	-0.27	-1.60*	1.37	-1.90*	0.41	-0.79	1.41*
dlWM	-0.55	-1.98*	-2.15*	-1.32	-1.72*	-1.25*	-1.73*	-0.02	-1.98*	-0.15	-0.90*	0.83
dWM	0.38	-1.68*	-1.59*	-1.47*	-0.38	-1.82*	-1.80*	-1.09	-1.32*	-0.29	-0.78	0.51
vGM	-1.93*	-1.34	-1.48	-1.15	-1.44	-1.54*	-1.76*	-1.35	-2.53*	-0.53	-0.78	-0.35
dGM	-2.26*	-1.54*	-1.74*	-1.31*	-0.11	-1.83*	-1.59*	-1.93*	-0.48	-1.04	-0.89	-1.02

The table includes the effect size for the comparison of each DTI parameter between G93A-SOD1 mice at 10 and 17 weeks of age. Statistically significant differences (p < 0.05) are marked by asterisks (*).

Unexpected statistically significant differences in FA, MD and AD were found by protocol A between WT-SOD1 mice at 10 and 17 weeks of age in several ROIs. On the contrary, protocol B showed only significantly different MD and RD values in one ROI, and protocol C showed no statistically significant difference (Table 3).

**Table 3:** Differences in DTI parameters of WT-SOD1 mice between 10 and 17 weeks of age.

ROI	Protocol A				Protocol B				Protocol C			
	FA	MD	AD	RD	FA	MD	AD	RD	FA	MD	AD	RD
vWM	1.99*	1.06	2.06*	-0.15	1.25	-0.80	0.16	-1.26*	-0.12	-0.33	-0.44	-0.09
vlWM	1.15	1.45*	2.49*	0.21	0.98	-0.97	-0.07	-1.10	-0.11	-0.02	-0.10	0.04
dlWM	0.68	1.15	1.70*	0.42	0.95	-1.20	-0.82	-1.18	-0.22	0.17	0.00	0.20
dWM	0.75	1.09	1.51*	0.49	0.46	-1.03*	-0.54	-0.97	-0.03	-0.11	-0.09	-0.06
vGM	2.32*	1.36*	1.84*	0.96	0.21	-0.75	-0.64	-0.78	0.01	0.02	-0.02	0.04
dGM	2.02*	1.40*	1.78*	0.92	-0.03	-0.87	-0.87	-0.78	0.72	-0.17	0.16	-0.39

The table includes the effect size for the comparison of each DTI parameter between WT-SOD1 mice at 10 and 17 weeks of age. Statistically significant differences (p < 0.05) are marked by asterisks (*).

### Histology and TEM

**Fig 6:**
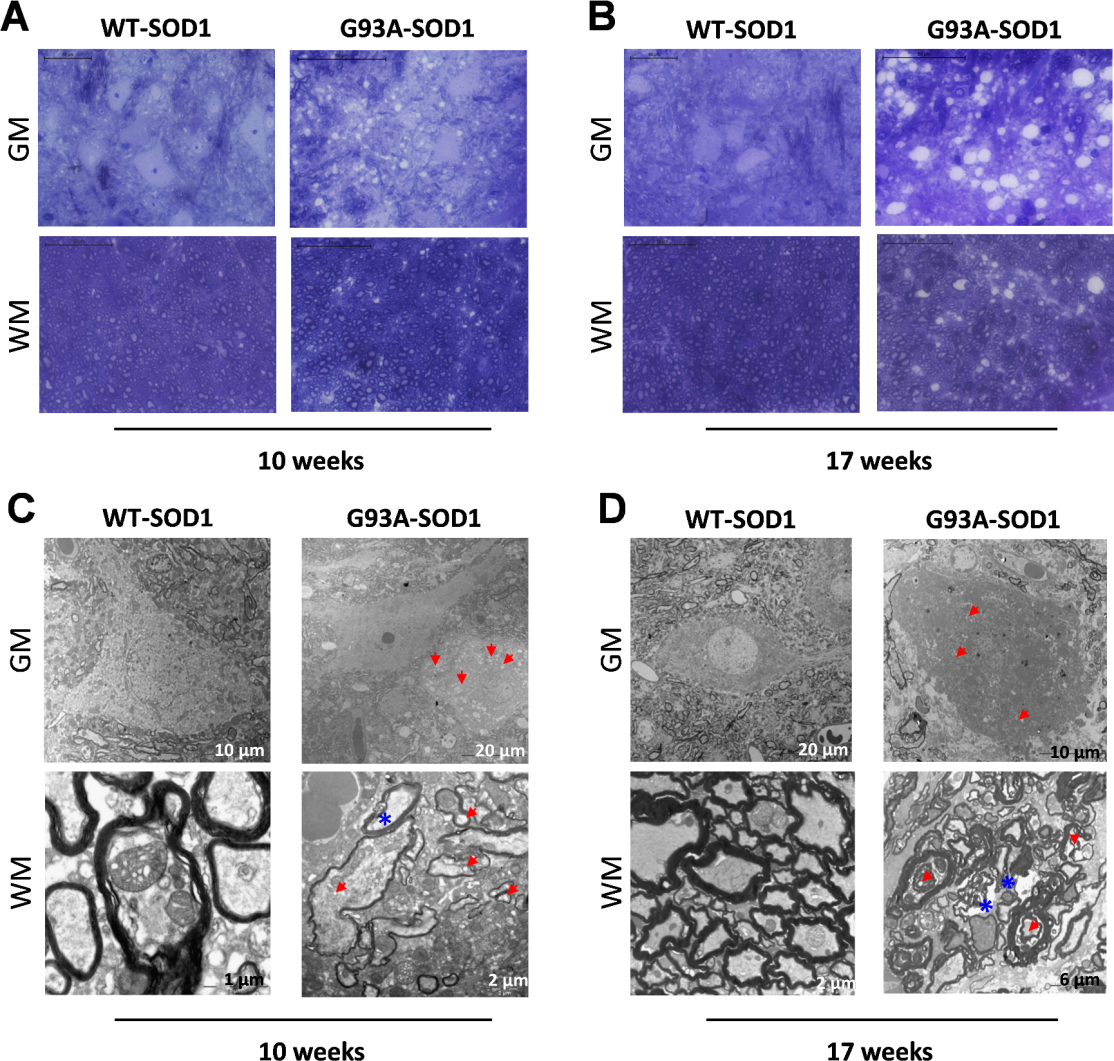
Toluidine staining and electron microscopy of lumbar spinal cord of WT-SOD1 and G93A-SOD1 mice. A) and B): macroscopic view of toluidine stained α-motor neurons (first row) and motor fibers (second row) in WT-SOD1 and G93A-SOD1 spinal cord at weeks 10 (A) and 17 (B). Scale bar = 50 μm. C) and D) Electron microscopy. The images show the presence of vacuoles (red arrows) in the soma (first row) and in the big axons (second row) of lumbar motoneurons of G93A-SOD1 mice at 10 (C) and 17 (D) weeks of age compared to age-matched WT-SOD1 mice. The images show also degenerated axons with disorganized myelin sheaths (blue asterisks) in G93A-SOD1 animals at 10 and 17 weeks of age compared to WT-SOD1 mice at the same age.

Histological and electron microscopy analysis showed evident motoneuron and axonal degeneration since 10 weeks of age in G93A-SOD1 mice compared to age-matched WT-SOD1 animals. Extensive vacuolization phenomena were found more prominent in motoneuron somas and in myelinated big axons in diseased animals at 17 weeks of age. TEM examination in motor spinal cord region showed completely degenerated axons with diffuse content and disorganized myelin sheaths in G93A-SOD1 animals compared to control mice at week 17 (Fig 6).

## Discussion

In the present work we compared three different dMRI protocols for the *in vivo* microstructural characterization of GM and WM in the mouse spinal cord. To identify the best protocol, we first evaluated the variability of the derived DTI parameters in control animals and then the sensitivity of each protocol to identify the altered microstructural features of the spinal cord of G93A-SOD1 mice at pre- and symptomatic phase of disease compared to control animals.

The three protocols correspond to different sampling strategies for dMRI: protocol A probes water diffusion along a large number of directions; protocol C explores only two directions (parallel and perpendicular to the spinal cord) with multiple diffusion weightings; protocol B has an intermediate number of directions and a b-value higher than that of protocol A but lower than the maximum b-value in protocol C. Constraining the acquisition time to be approximately the same, there was the possibility to acquire multiple repetitions in protocols B and C as opposed to the single one in protocol A, given the lower number of volumes to acquire in each repetition. Moreover, we used higher b-values in protocols B and C than in A, since a sequence with more repetitions can afford a higher signal loss without excessively reducing the signal in the averaged images. We visually checked the results of the registration procedure, thus verifying that the SNR in the single repetitions was sufficient for motion correction.

### Mean Values of DTI Parameters

Protocol A provided higher values of all the DTI parameters compared to protocol B, and protocol B provided higher values than protocol C, with few exceptions. This can be explained considering the different acquisition parameters: FA is likely overestimated by protocol A because of the relatively low SNR [20,60,61], and underestimated by protocol C because two directions are insufficient for a good characterization of diffusion anisotropy. Lower diffusivities are estimated by the protocols with higher diffusion weightings [18,19,62].

### Inter- and Intra-Subject CV

For the subsequent analysis of measurement variability in controls, we chose to use the CV instead of the standard deviation of the measures because the considered DTI parameters have different orders of magnitude and even the same parameter estimated by different protocols can show biases in the mean value, as just pointed out. The normalization inherent in the calculation of the CV should remove these effects from the variability estimates and allow a direct comparison, identifying the best performing protocol in terms of measure reliability and reproducibility [29,33,58,63]. In particular, the inter-subject CV describes the variability among the ROI-averaged parameters estimated in the 7 mice, therefore accounting for individual differences, which are assumed to be low considering healthy age-matched animals, and for differences between acquisition sessions and due to uncertainty of DTI estimation, which are the focus of this analysis. The intra-subject CV refers to the variability inside the ROIs, caused only by image noise and estimation uncertainty, since the ROIs are assumed to be homogeneous.

Protocol B showed the overall lowest inter- and intra-subject CV, competing in some regions with protocol A for FA and with protocol C for diffusivities.

FA derivation relies on the knowledge of the relative angular distribution of water motions. It is therefore intuitive that, in a general context, a larger number of diffusion gradient directions brings to a more precise evaluation of anisotropy, whereas two directions only, as in protocol C, cannot provide a sufficient angular sampling. Nevertheless, the relationship between the number of directions and the accuracy of DTI parameters estimation is not straightforward and depends on many factors, such as the other acquisition parameters and the microstructural features of the tissue of interest [19,27,61]. Indeed, in an environment with a relatively simple structure, as is the case of spinal cord cylindrical geometry, adding directions may not give a remarkable contribution to the measure accuracy. In the present study, 12 directions seem to be enough for the purpose and protocol B repeatability is at least comparable to protocol A for all the considered metrics.

Diffusivity measures, on the other hand, do not depend on the angular resolution as much as they do on the b-value [64]. Protocol A, with the lowest diffusion weighting, provides a worse estimation of axial, radial and mean diffusivity with respect to the other protocols. Moreover, the fewer directions of protocols B and C allow for more averages/repetitions within the same acquisition time, leading to higher SNR in each averaged volume. The higher intra-subject variability shown by protocol A is likely related to this lower SNR.

### Angular Deviation

The angular deviation between the direction of the tensors and the slice direction was low and had little effect on diffusion parameters quantification. The difference between the MDs derived by the tensor and by its components along the axes of the imaging frame of reference was virtually zero and probably due to rounding errors only, as expected since MD can be equivalently computed from the diffusivities estimated along any set of three orthogonal directions [4]. For the other parameters this difference was relatively higher, but still almost negligible.

Higher angular deviations were measured in dorso-lateral regions than in the other WM ROIs. The anatomy of the spinal cord itself can help interpret this finding: large myelinated sensitive axons are added at each segment and, entering the dorsal column in a caudorostral order, they move close to the medial edge forming a stripe [65]**.**

The parameters estimated by protocol C had weak correlations with the deviation angle amplitude, probably because the variability between animals was higher than the effect of the angular approximation. Thus, the bad performance of protocol C should not be mainly related to the imprecision in setting the geometry acquisition. We found only an unexpected positive correlation for MD, though weak, probably due to an unbalanced increase in RD with respect to the relative decrease in AD.

### Differences between G93A-SOD1 and WT-SOD1 Mice

At 10 weeks of age we found only few significant differences in DTI parameters between pathological and healthy animals, and only with protocol B this difference was compatible with the later findings. This is in agreement with previous studies that reported alterations on MRI only after the 12^th^ week in this ALS murine model [14]. However, TEM showed modifications in motor neurons of G93A-SOD1 spinal cord already at 10 weeks of age. Spinal cord dMRI sensitivity is probably limited by its spatial resolution, not allowing the specific analysis of the different sensitive and motor tracts that could have allowed detecting the early motor neuron damage, limited to motor tracts.

Assumed that the evident disease expression highlighted by histology and TEM at 17 weeks is recognizable by MRI, we assessed the sensitivity of the protocols in detecting significant structural differences between ALS animals and controls by comparing the results obtained in the two groups at that age.

Protocols A and B were more sensitive than protocol C in differentiating between G93A-SOD1 and WT-SOD1 mice: protocol A provided a better discrimination than protocol B for MD, whereas protocol B was slightly better for FA and RD.

As expected [14,30], FA and AD are reduced as a consequence of the fiber disorganization shown by our TEM results. The unexpected reduction of MD, detected with all the three protocols, could be explained by the vacuolization phenomena observed in G93A-SOD1 motor neuron by our histology and TEM as well as in previous studies [66]; indeed vacuolization is associated with diffusivity reduction in other neurodegenerative pathologies such as Creutzfeldt-Jakob disease [67,68]. Another possible cause of MD reduction might be the abnormal accumulation of superoxide dismutase 1 (SOD1) protein and intermediate filaments in the intra- and extra-cellular space [69,70]. RD shows different trends in the three protocols, suggesting a more complex underlying scenario: the balance between an increase, due to fiber degeneration, and a reduction, due to vacuolization or proteins and filaments accumulation. The different results of the three protocols probably depend on their different sensitivity to these opposite mechanisms.

### Monitoring the Pathological Changes over Time

The pattern of differences in DTI parameters between G93A-SOD1 animals over time (10 vs. 17 weeks of age) is similar to that found between G93A-SOD1 and WT-SOD1 mice at 17 weeks, but the differences themselves are smaller. This finding suggests that the pathological alterations in G93A-SOD1 animals at 10 weeks of age result indeed in a variation of diffusion parameters, though not enough evident to be reliably highlighted by MRI, and the difference between DTI parameters estimated in mice at 10 and 17 weeks can be considered as a marker of disease progression as shown in a previous study [14].

In the detection of such a difference protocol C showed the overall best performance for FA, whereas protocol A and B were much better than C to highlight changes in MD and especially AD over time, and A was slightly better than the others for RD.

Unexpectedly, protocol A showed also significant differences between WT-SOD1 animals at 10 and 17 weeks of age. Conversely, protocols B and C found almost no difference, as expected since mice are already adult at 10 weeks and important changes in the microstructure of healthy spinal cord do not likely occur in the considered period of time. Moreover, FA apparently increased according to protocol A, which is not plausible for ageing mice. All the significant differences are opposite to those found in G93A-SOD1, and thus are likely responsible for a factitious component of the differences found by protocol A between G93A-SOD1 and WT-SOD1 animals at 17 weeks. Consequently, these coherent changes over time should depend on the different operating conditions of the two experimental sessions (slightly different position of the mouse in the scanner, different state of the gradient coils or other hardware components). Protocol A is apparently more sensitive to these external conditions and the derived results seem less reproducible and reliable. On the contrary, results from protocols B and C seem more robust.

### Comparison with Literature Results

The optimal acquisition parameters identified in this study confirm largely accepted recommendations for brain and spinal dMRI, but highlight that a moderate number of diffusion directions is sufficient in the case of the SC. This can explain the good results obtained by mouse SC DTI studies applying only 6 directions to limit the acquisition time in spin echo sequences. Conversely, according to our investigation 2 directions are not sufficient for a good characterization of the SC, contrary to what reported by Tu et al. [49].

Our dMRI results on the comparison between G93A-SOD1 and WT-SOD1 mice are generally in accordance with the few previous works found in literature [13,14], but we obtained significant differences in all the WM areas and not only in the ventral ones as previously reported, and also in GM, which was not considered [14] or did not provide significant results [13] in previous studies. Thus we could speculate that our optimal protocol is more sensitive to microstructural changes in all the SC areas; however, we have to point out that it would be pretentious to claim that our protocol is optimal for any possible condition, and a different sampling scheme might be required depending on a project’s aims.

### Limitations and Future Work

A few limitations of this study have to be highlighted. First of all, the three considered protocols are different both in terms of number of directions and b-value. A more comprehensive approach should have compared protocols varying each parameter a time; however, the inclusion of a higher number of different protocols would have increased the total acquisition duration over the allowed time for a single session. Acquiring the same animals in multiple sessions was unfeasible for the diseased mice, and testing additional protocols on different groups would have added a source of bias or variability. For this reason we decided to test only three protocols for a preliminary exploration of dMRI performance in the mouse spinal cord. We will perform a finer analysis in future works, starting from the results of this study; in particular, we will explore imaging schemes with intermediate numbers of directions between 12 and 126 and with b-values of at least 1000 s/mm^2^.

Another issue is the assumption that the spinal cord is parallel to the slice direction, which is only approximately true. Besides the effect on the parameters estimated by protocol C, which we showed to be low, this could influence also the measures from the other protocols, since the voxel is not isotropic and a deviation from the spine direction would cause partial volume effects. These concerns would be more important if studying the whole spinal cord.

At last, perhaps the most important issue in diffusion studies, especially when the task is the comparison of imaging methods for the characterization of biological tissues, is the lack of a ground truth to validate the results. In our study we compared the protocols assuming that the best performance is associated to lowest variability in controls and greatest differentiation between healthy and pathological mice; similarly, in the analysis of angular deviation we assumed that the principal direction identified by DTI coincides with fiber direction. A possible approach to understand the reliability of the measures obtained with the different protocols would have been to perform numeric simulations, but this would have required a more complete a priori knowledge of the microstructural features of the spinal cord relevant for diffusion studies. In future works, simulations could be applied for an a priori comparison of acquisition protocols, but in vivo experiments are necessary to validate the relevance of the findings, especially in pathological conditions.

Interestingly, for the first time we demonstrated a significant decrease of AD in ALS mice compared to control mice at 10 weeks. A further study will be dedicated to deepen the investigation at early time points, to better understand if DTI parameters can be reliable biomarkers of early neurodegeneration. Another direction for future work could be the assessment of the optimal acquisition scheme in other SC diseases. Indeed, if the pathological mechanisms and the anatomical structures of interest are different from those investigated here, a proper tuning of acquisition parameters would be necessary.

### Conclusion

Our tests pointed out that for the specific purpose of spinal cord imaging, a diffusion tensor scheme with a relatively high b-value and a relatively low number of diffusion-encoding gradient directions is optimal. With a constraint on the acquisition time, a protocol with a higher number of directions proved to be similarly effective in the detection of disease, but less reproducible and more prone to noise. The optimal protocol allowed a good differentiation between ALS and healthy mice, suggesting a possible relevant role of dMRI in the diagnostic approach to motor neuron diseases. Our results could have important implications for the optimization of SC dMRI in preclinical and clinical studies of ALS and other diseases, though a further tuning of acquisition parameters would be required given the specific conditions in each scenario.

## Supporting Information

S1 Fig FA comparison between G93A-SOD1 and WT-SOD1 mice. The bar graphs show the FA values estimated by each protocol (Prt), averaged among 7 WT-SOD1 (white) and 7 G93A-SOD1 mice (gray), at 17 weeks of age. Mean values (± standard deviation) are shown for a different ROI in each panel. Significant differences (p < 0.05) between G93A-SOD1 and WT-SOD1 are labeled with asterisks.
(TIF)

S2 Fig MD comparison between G93A-SOD1 and WT-SOD1 mice. The bar graphs show the MD values estimated by each protocol (Prt), averaged among 7 WT-SOD1 (white) and 7 G93A-SOD1 mice (gray), at 17 weeks of age. Mean values (± standard deviation) are shown for a different ROI in each panel. Significant differences (p < 0.05) between G93A-SOD1 and WT-SOD1 are labeled with asterisks.
(TIF)

S3 Fig AD comparison between G93A-SOD1 and WT-SOD1 mice. The bar graphs show the AD values estimated by each protocol (Prt), averaged among 7 WT-SOD1 (white) and 7 G93A-SOD1 mice (gray), at 17 weeks of age. Mean values (± standard deviation) are shown for a different ROI in each panel. Significant differences (p < 0.05) between G93A-SOD1 and WT-SOD1 are labeled with asterisks.
(TIF)

S4 Fig RD comparison between G93A-SOD1 and WT-SOD1 mice. The bar graphs show the RD values estimated by each protocol (Prt), averaged among 7 WT-SOD1 (white) and 7 G93A-SOD1 mice (gray), at 17 weeks of age. Mean values (± standard deviation) are shown for a different ROI in each panel. Significant differences (p < 0.05) between G93A-SOD1 and WT-SOD1 are labeled with asterisks.
(TIF)

S1 Table CNR of the maps of DTI metrics. The table reports the CNR between WM and GM measured in the map of each DTI metric from each of the three acquisition protocols.
(DOCX)

S2 Table Angular deviations. The table shows the angle (in degrees) between the slice direction and the principal diffusion direction estimated by protocols A and B in WT-SOD1 mice in each region at each week.
(DOCX)

S3 Table Errors associated with angular deviations. The table lists the difference between the parameters estimated by protocol A in WM ROIs of WT-SOD1 mice from the eigenvalues of the diffusion tensor and from its components along the axes of the imaging frame of reference. The MD variation was always less than 10^−15^. Protocol B provided similar differences (not shown).
(DOCX)
