# Supplementary figures and images for: Comparison of Diffusion MRI Acquisition Protocols for the In Vivo Characterization of the Mouse Spinal Cord: Variability Analysis and Application to an Amyotrophic Lateral Sclerosis Model
Source: PLoS One. 2016 Aug 25;11(8):e0161646. doi: 10.1371/journal.pone.0161646 (PMC4999133; doi:10.1371/journal.pone.0161646)

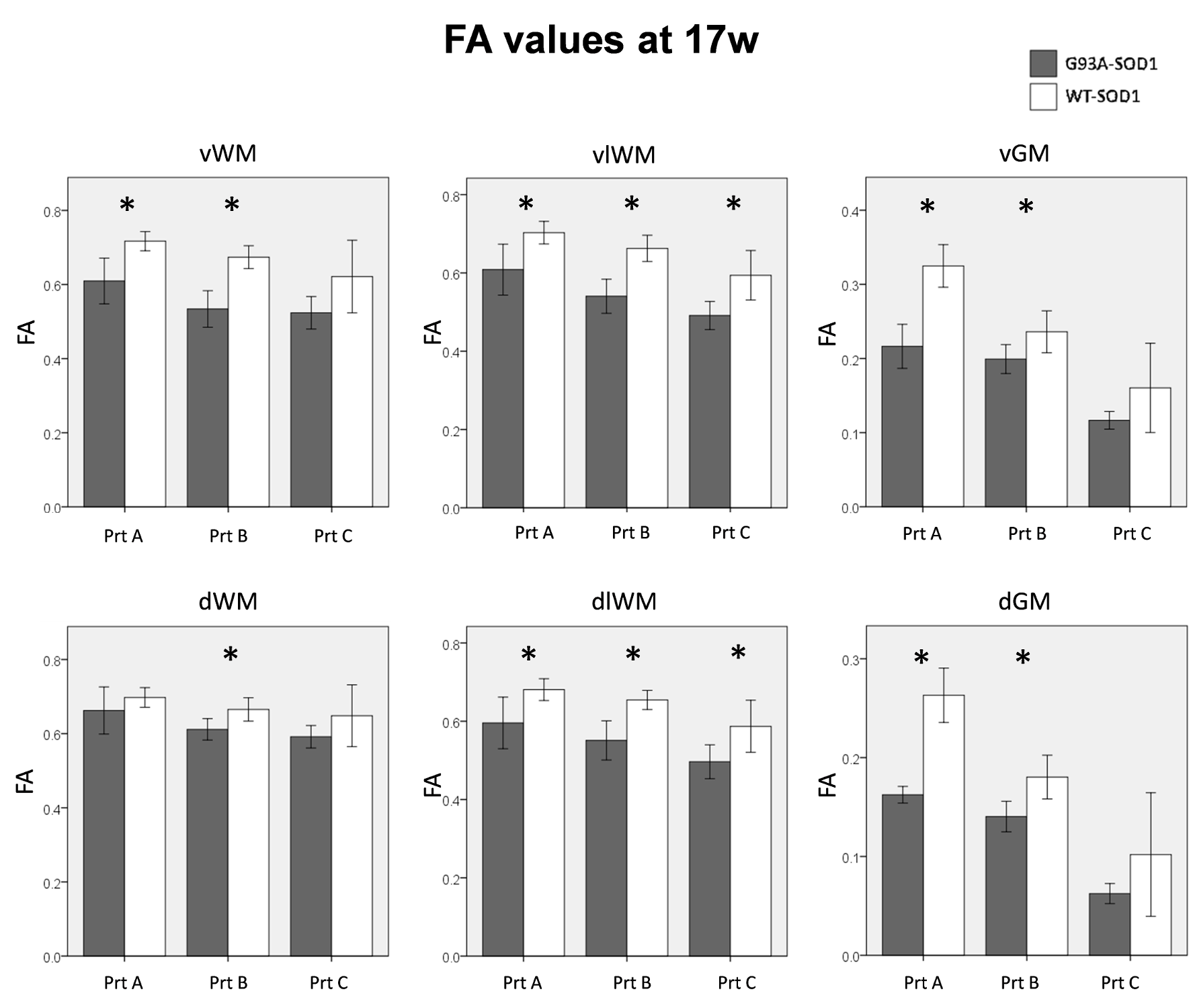

Supplement: S1 Fig — The bar graphs show the FA values estimated by each protocol (Prt), averaged among 7 WT-SOD1 (white) and 7 G93A-SOD1 mice (gray), at 17 weeks of age. Mean values (± standard deviation) are shown for a different ROI in each panel. Significant differences (p < 0.05) between G93A-SOD1 and WT-SOD1 are labeled with asterisks. (TIF) [file pone.0161646.s001.tif]

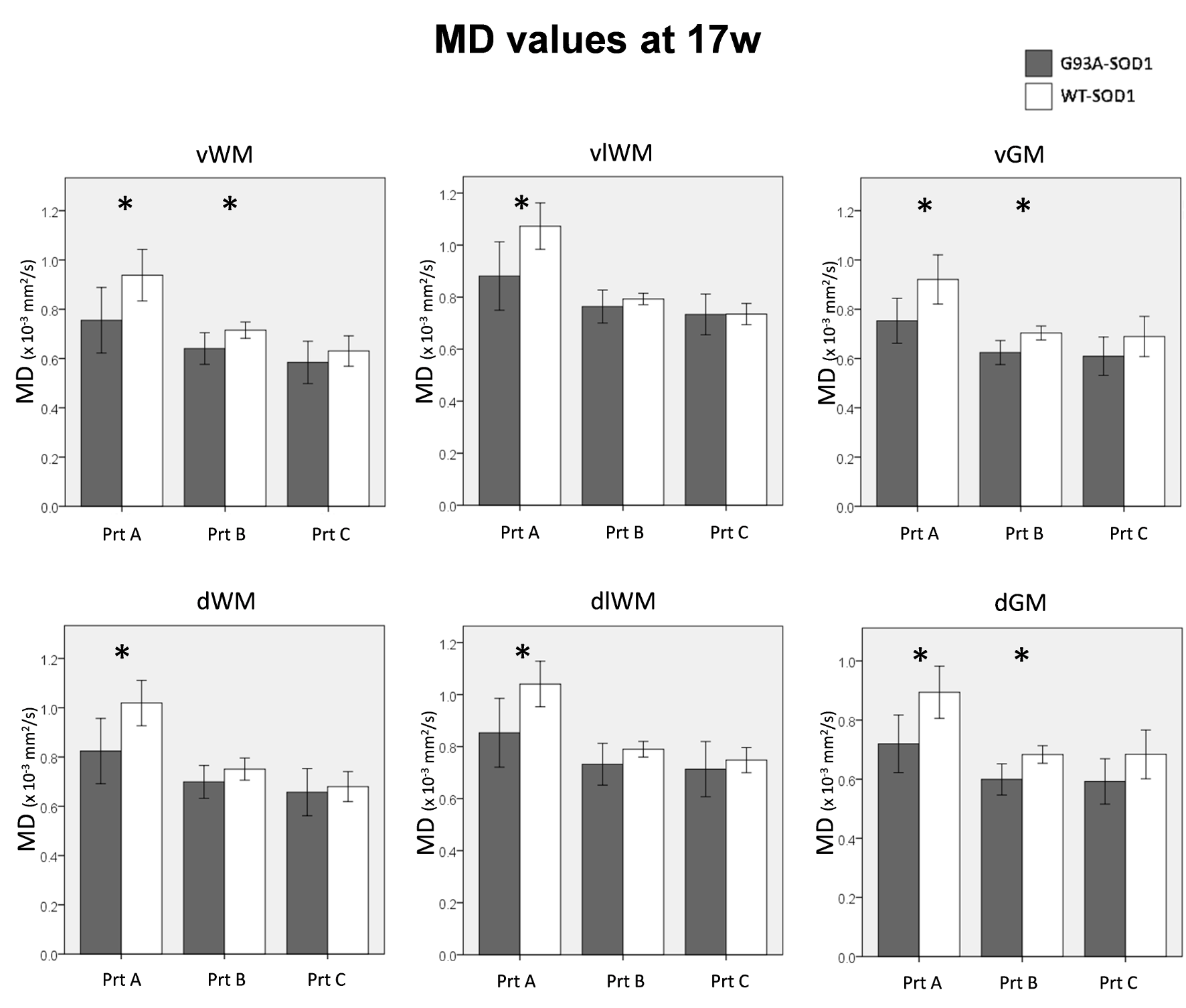

Supplement: S2 Fig — The bar graphs show the MD values estimated by each protocol (Prt), averaged among 7 WT-SOD1 (white) and 7 G93A-SOD1 mice (gray), at 17 weeks of age. Mean values (± standard deviation) are shown for a different ROI in each panel. Significant differences (p < 0.05) between G93A-SOD1 and WT-SOD1 are labeled with asterisks. (TIF) [file pone.0161646.s002.tif]

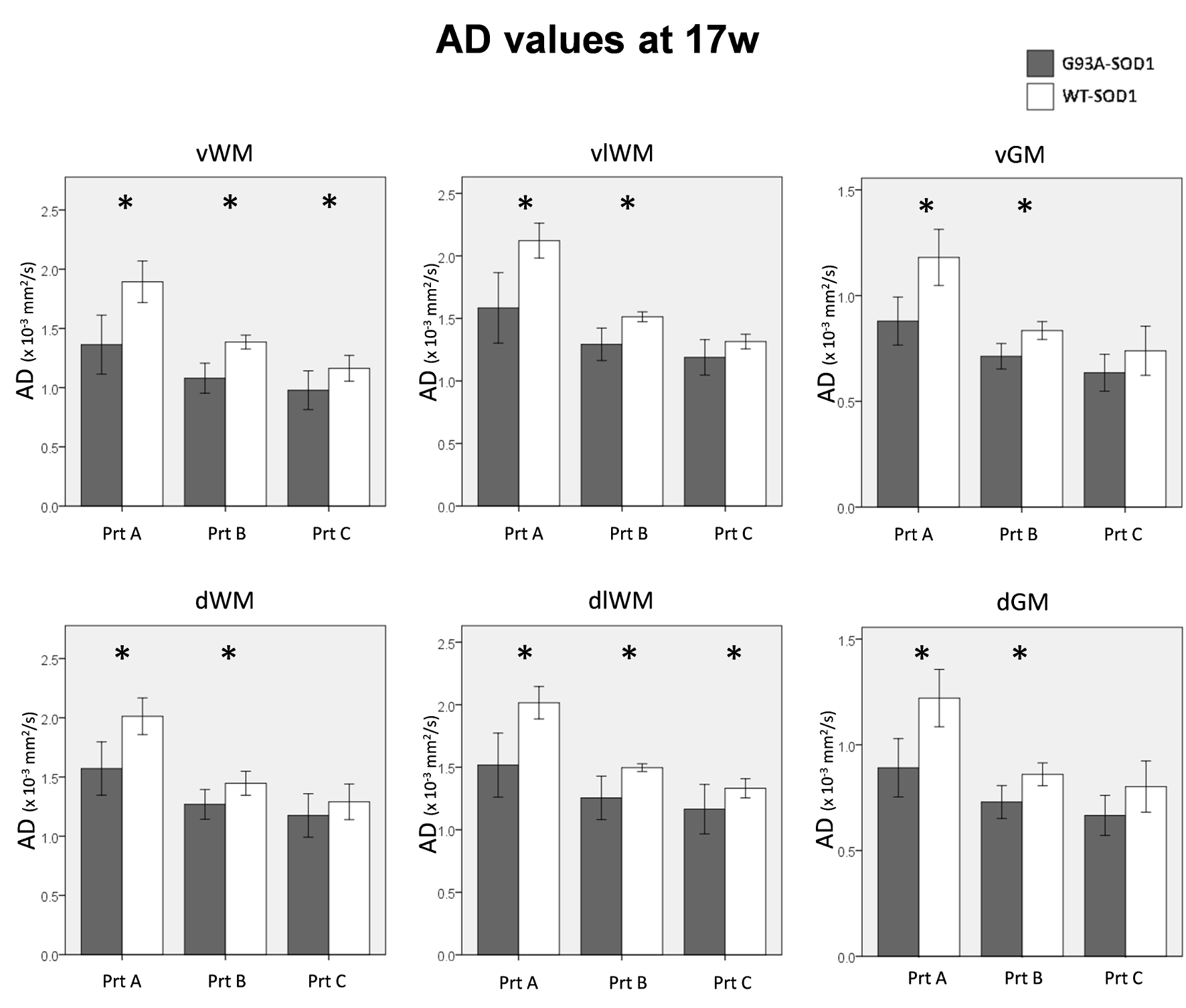

Supplement: S3 Fig — The bar graphs show the AD values estimated by each protocol (Prt), averaged among 7 WT-SOD1 (white) and 7 G93A-SOD1 mice (gray), at 17 weeks of age. Mean values (± standard deviation) are shown for a different ROI in each panel. Significant differences (p < 0.05) between G93A-SOD1 and WT-SOD1 are labeled with asterisks. (TIF) [file pone.0161646.s003.tif]

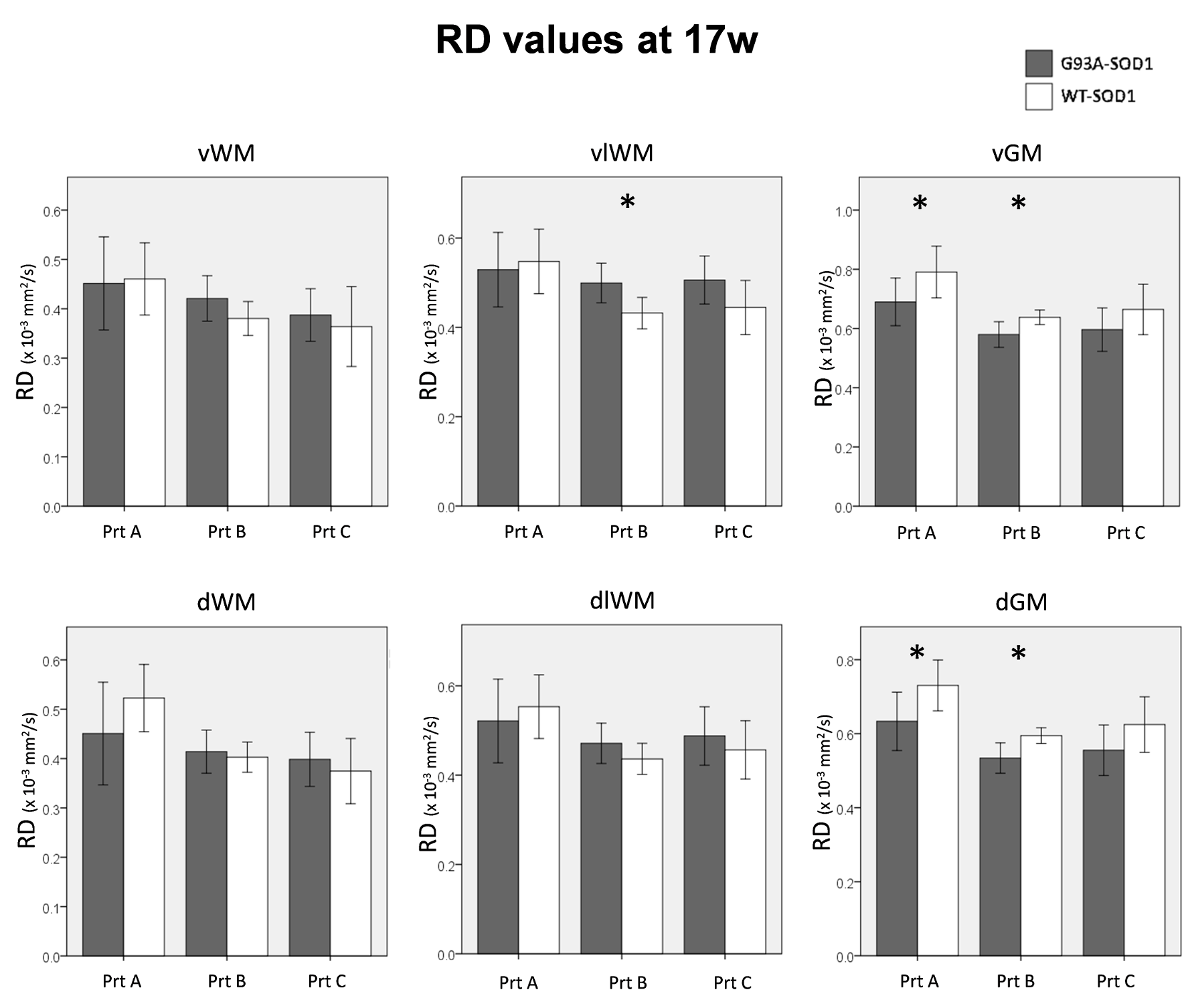

Supplement: S4 Fig — The bar graphs show the RD values estimated by each protocol (Prt), averaged among 7 WT-SOD1 (white) and 7 G93A-SOD1 mice (gray), at 17 weeks of age. Mean values (± standard deviation) are shown for a different ROI in each panel. Significant differences (p < 0.05) between G93A-SOD1 and WT-SOD1 are labeled with asterisks. (TIF) [file pone.0161646.s004.tif]
