# Supplementary material for: Comparison of Diffusion MRI Acquisition Protocols for the In Vivo Characterization of the Mouse Spinal Cord: Variability Analysis and Application to an Amyotrophic Lateral Sclerosis Model
Source: PLoS One. 2016 Aug 25;11(8):e0161646. doi: 10.1371/journal.pone.0161646 (PMC4999133; doi:10.1371/journal.pone.0161646)
Supplement: S1 Table — The table reports the CNR between WM and GM measured in the map of each DTI metric from each of the three acquisition protocols. (DOCX) [file pone.0161646.s005.docx]

**S1 Table: CNR of the maps of DTI metrics**

|  | Protocol A | Protocol B | Protocol C |
| --- | --- | --- | --- |
| FA | 4.53 | 5.55 | 5.48 |
| MD | 0.95 | 1.01 | 0.35 |
| AD | 3.07 | 3.97 | 3.76 |
| RD | -1.43 | -2.66 | -2.70 |

The table reports the CNR between WM and GM measured in the map of each DTI metric from each of the three acquisition protocols.
