# Supplementary material for: Comparison of Diffusion MRI Acquisition Protocols for the In Vivo Characterization of the Mouse Spinal Cord: Variability Analysis and Application to an Amyotrophic Lateral Sclerosis Model
Source: PLoS One. 2016 Aug 25;11(8):e0161646. doi: 10.1371/journal.pone.0161646 (PMC4999133; doi:10.1371/journal.pone.0161646)
Supplement: S2 Table — The table shows the angle (in degrees) between the slice direction and the principal diffusion direction estimated by protocols A and B in WT-SOD1 mice in each region at each week. (DOCX) [file pone.0161646.s006.docx]

**S2 Table: Angular deviations**

| ROI | Prt A | | Prt B | |
| --- | --- | --- | --- | --- |
|  | 10w | 17w | 10w | 17w |
| vWM | 8.80 | 10.53 | 8.32 | 7.72 |
| vlWM | 9.46 | 10.77 | 9.46 | 8.80 |
| dlWM | 13.76 | 13.89 | 13.75 | 12.77 |
| dWM | 8.21 | 8.06 | 7.03 | 6.75 |
| vGM | 54.62 | 36.91 | 47.43 | 48.12 |
| dGM | 30.85 | 22.05 | 27.48 | 30.13 |

The table shows the angle (in degrees) between the slice direction and the principal diffusion direction estimated by protocols A and B in WT-SOD1 mice in each region at each week.
