# Supplementary material for: Comparison of Diffusion MRI Acquisition Protocols for the In Vivo Characterization of the Mouse Spinal Cord: Variability Analysis and Application to an Amyotrophic Lateral Sclerosis Model
Source: PLoS One. 2016 Aug 25;11(8):e0161646. doi: 10.1371/journal.pone.0161646 (PMC4999133; doi:10.1371/journal.pone.0161646)
Supplement: S3 Table — The table lists the difference between the parameters estimated by protocol A in WM ROIs of WT-SOD1 mice from the eigenvalues of the diffusion tensor and from its components along the axes of the imaging frame of reference. The MD variation was always less than 10−15. Protocol B provided similar differences (not shown). (DOCX) [file pone.0161646.s007.docx]

**S3 Table: Errors associated with angular deviations**

| ROI | FA variation | | MD variation | | AD variation | | RD variation | |
| --- | --- | --- | --- | --- | --- | --- | --- | --- |
|  | 10w | 17w | 10w | 17w | 10w | 17w | 10w | 17w |
| vWM | -2.99% | -2.40% | 0.00% | 0.00% | -1.70% | -2.11% | 2.80% | 3.70% |
| vlWM | -5.05% | -4.12% | 0.00% | 0.00% | -2.19% | -2.59% | 3.71% | 4.62% |
| dlWM | -8.93% | -8.65% | 0.00% | 0.00% | -4.66% | -4.69% | 7.67% | 7.65% |
| dWM | -3.27% | -2.94% | 0.00% | 0.00% | -1.37% | -1.55% | 2.21% | 2.43% |

The table lists the difference between the parameters estimated by protocol A in WM ROIs of WT-SOD1 mice from the eigenvalues of the diffusion tensor and from its components along the axes of the imaging frame of reference. The MD variation was always less than 10^-15^. Protocol B provided similar differences (not shown).
